# Structure Performance Correlation of N-Heterocyclic Oligomer Leveler for Acid Copper Plating of Advanced Interconnects

**DOI:** 10.3390/molecules28062783

**Published:** 2023-03-20

**Authors:** Chuan Peng, Yuehui Zhai, Xianming Chen, Chong Wang, Yan Hong, Yuanming Chen, Wei He, Guoyun Zhou, Binyun Liu

**Affiliations:** 1 School of Materials and Energy, University of Electronic Science and Technology of China, Chengdu 610054, China; 2 Jiangxi Institute of Electronic Circuits, Pingxiang 337009, China; 3 Zhuhai ACCESS Semiconductor Co., Ltd., Zhuhai 519099, China; 4 Guangdong Key Laboratory of Enterprises on Electronic Chemicals, Guangdong Guanghua Tech. Co., Ltd., Zhuhai 515063, China

**Keywords:** copper electroplating, oligomer, throwing power, leveler

## Abstract

Levelers, as an essential part of organic additives in copper electroplating, play a crucial role in the fabrication of sophisticated interconnects in integrated circuits, packaging substrates, and printed circuit boards. In this work, four N-heterocyclic oligomers were synthesized and characterized, along with investigations of their electrochemical behaviors and their synergism with other bath components. The corresponding effects of the oligomers on the deposited copper films were analyzed by morphological and compositional characterizations. The leveling mechanism of the oligomers was further discussed with the aid of quantum chemical calculations. The results exhibit that each of these N-heterocyclic oligomers holds a particular degree of leveling ability. The oligomer of 1,3-bis(1-imidazolyl)propane and 1,3-dichloro-2-propanol (IPIEP) is the best leveler for THs plating compared with the other three oligomers. It was found that the hydroxyl group in IPIEP enhances the hydrophilicity of the modified molecule and triggers a more stable complexation between IPIEP and H_2_O−Cu(I)−MPS. Moreover, imidazole demonstrates a better practicality than piperazine. This work recommends the combination of N-heterocycles in planar conformation with modification by the hydroxyl group to synthesize high-performance straight-chain levelers.

## 1. Introduction

Fifth-generation communication promotes the development of electronic interconnects with a high integration, high bandwidth, and high frequency transmission. Copper electroplating technology is the primary method for forming electronic interconnection in integrated circuits (ICs), packaging substrates, and printed circuit boards (PCBs), which plays a crucial role in the booming electronics industry [1,2,3].

Under the synergistic effects of organic additives (brighteners, suppressors, and levelers), electroplated copper is uniformly deposited on the inner wall of through holes (THs) as the conformal layer or micro-through-hole filler [3]. The performance of electroplating copper film in THs is decided by the shape of holes, electrolyte formula, plating current, solution convection, etc. [4,5]. The conformal deposition in THs has been explained by the theory of secondary current distribution, but the via filling was considered abnormal and termed as super filling due to its behavior [6,7]. Moffat proposed and then revised the curvature-enhanced adsorbate coverage (CEAC) model based on the growth process, revealing the accelerating effect of the brightener in the bottom-up growth of vias [6,7]. Later, Dow discovered the convection-dependent property of levelers [8], and Broekmann found the N-type negative differential resistance (NDR) phenomenon in the bath solution [9]. These findings have established a systematic via-filling theory and emphasized the importance of levelers in copper electroplating.

Typically, the suppressor is chosen from PEG or its derivatives [10,11,12], and the brightener is sodium bis-(3-Sulfopropyl)disulfide (SPS) or sodium 3-mercapto propane sulphonate (MPS) [8,13]. In contrast, there are a few levelers with quite different leveling performances [14,15,16,17] that are applied to various plating formulas to meet the multifarious demands of interconnecting manufacture. High-performance levelers, therefore, play an essential role in the commercial plating formula. Levelers were generally selected from dyes in the early days of copper electroplating, such as Janus Green B [18,19], a classic one in the literature [19,20,21]. Early work suggested that the leveling ability of JGB originated from the electrochemical reduction and cleavage of its −N=N− group at the tip or protrusion of the cathode surface [22]. However, this hypothesis implies a large amount of JGB consumption and low current efficiency of copper deposition, which is not consistent with the actual situation.

Besides dye-based levelers, in recent years, organic oligomer levelers (nitrogen-containing heterocycles chained by linker groups) have attracted increasing attention in research and application [15,23,24]. Due to the unclear structure–property relationships of these oligomers, the need to develop new oligomer leveler machines with a high through-hole filling performance is very time-consuming and challenging. Several research groups have started to purposefully design various oligomer levelers to investigate their structure–property relationships in THs filling. Quaternary ammonium salts have been systematically studied because of their excellent via filling properties [25,26]. Broekmann used polymerizates of imidazole and epichlorohydrin (IMEP) as a model leveler to study the oscillatory behavior during electrodeposition and concluded that the leveling effect originates from the coordination between Cu(I) and the leveler, in which the hydroxyl functional group coordinates Cu(I) to form the IMEP−Cu(I)−MPS complex, inhibiting the activity of MPS [27]. In contrast, Bandas claimed that the activity of MPS is inhibited by electrostatic pairing with the N-heterocyclic leveler and the deposition of copper ions is occluded by the steric chemical structure of the oligomers [28].

To develop a high-performance leveler, it is significant to reveal the relationship between the chemical composition of levelers and the leveling properties. The interfacial electrochemical mechanism also needs elucidation. In this work, a series of oligomers involving N-heterocyclic groups and O-functional groups via methylene links were prepared and characterized to study the structure–performance correlation of levelers. The electrochemical behavior of these oligomers in the plating bath and their interactions with other additive components were also investigated. The effects on the microstructure and surface morphology of the electrodeposited copper were characterized. Quantum chemical calculations and the THs plating results were combined to propose the probable mechanism of these leveling candidates. This work further promotes the theoretical study of levelers based on Broekmann and Schmidt’s theory, providing a possible path to advanced levelers.

## 2. Results and Discussion

### 2.1. Electrochemical Characterization

**Figure 1:**
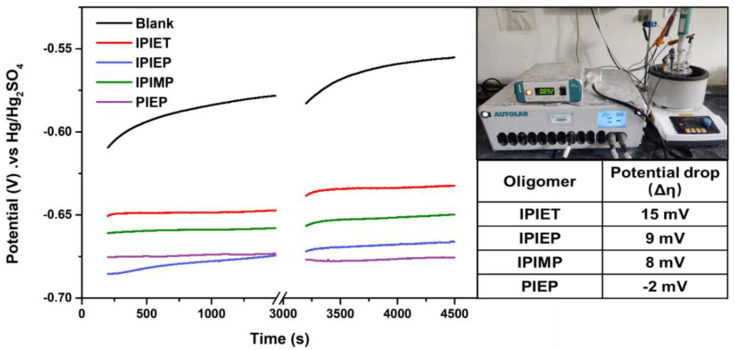
GMs of various oligomer-containing solutions at a current density of 1.5 A/dm^2^. The rotation speeds of Cu-RDE are 2500 rpm (0~1500 s) and 200 rpm (3000~4500 s). Each bath is composed of base electrolyte, 5 g/L PEG, 10 mg/L SPS, and 5 mg/L oligomer, respectively. The insets show the instrument and the values of potential drops.

Figure 1 illustrates that these four oligomers exhibit different antagonistic effects on electroplating. Based on the results of GMs, all four oligomers inhibit the accelerator’s activity, but PIEP fails to present a leveling ability. Each of the oligomers is stable under electroplating conditions without decomposition (Figure S2). Different rotation speeds (2500 rpm and 200 rpm) are operated to simulate the mass transfer behavior in the regions of the board surface (2500 rpm) and the center (200 rpm) of the micro-holes, respectively [18]. The cathode deposition potential (η) is thereby correlated with rotation speeds. The formula defining the difference in cathode potentials—Δη = η_200 rpm_ − η_2500 rpm_—is often taken as the indicator to evaluate the leveling ability [8]. When the Δη is higher, the intensive convection-dependent polarization is stronger [8].

Compared to the GM curves with the addition of a suppressor and accelerator, the cathodic potential decreases significantly with the injection of 5 mg/L oligomers, which implies that these four oligomers all effectively inhibit the activity of the accelerator. Except for PIEP, the cathodic steady potential value of the oligomers follows the order of IPIEP > IPIMP > IPIET in both fast and low-rotation speed regions. According to this trend, the hydroxyl-modified oligomer (IPIEP) has the strongest inhibition ability compared to the ethoxy (IPIET) and carbonyl group (IPIMP). Moreover, the Δη from IPIET, IPIMP, and IPIEP are all positive, pointing out that the copper deposition rate is negatively correlated to the strength of the forced convection [21], which predicts that these “base electrolyte + SPS + PEG + oligomer” formulas may achieve a conformal plating of THs and bottom-up super-filling of the micro-vias.

**Figure 2:**
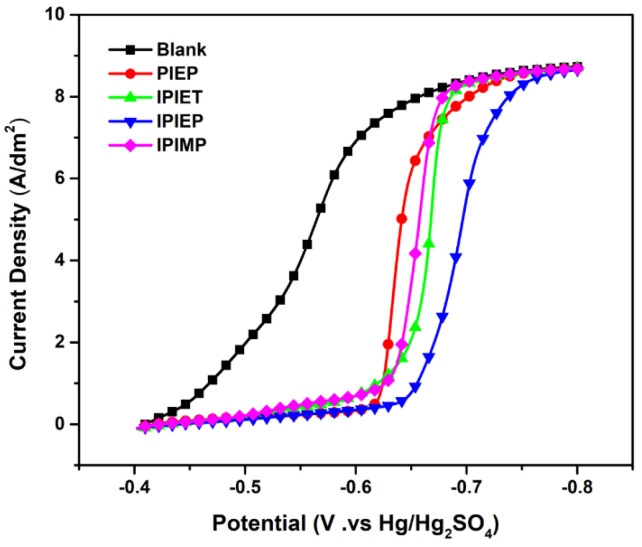
Potentiodynamic polarization curves related to 5 mg/L oligomers in the base electrolyte. The electrode rotation speed is 1500 rpm.

The polarization curves suggest that IPIEP shows the strongest inhibition in the electrolyte without a suppressor and an accelerator. To further analyze the effects of different functional groups and N-heterocyclic groups on their inhibitory capacity, the potentiodynamic polarization curves of the four oligomers were measured (Figure 2). With the injection of oligomers, the decaying current shifts negatively, occurring at −0.41 V in the bath, which may be attributed to the adsorption of oligomers on the copper surface. As shown in Figure 2, IPIEP has the strongest inhibiting capability (−0.65 V) compared with the other oligomers. Notably, the order of the start-up cathodic potential (IPIEP > IPIET > IPIMP) does not follow the order of the cathodic steady potential (Figure 1), which implies that the different functional groups are involved in the electrochemical behaviors of oligomers. The hydroxyl group enhances the effect of cathodic polarization more significantly than the other groups. Because of the differences in the spatial configuration and charge distribution between piperazine and imidazole, PIEP exhibits the weakest inhibiting performance (−0.61 V). The differences between piperazine and imidazole are discussed in Section 3.3 in detail.

### 2.2. Electroplating

**Figure 3:**
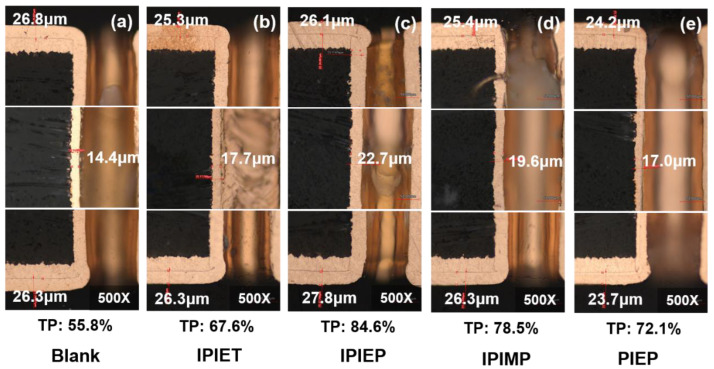
Cross-section metallographic photos of THs obtained from the base electrolyte with 500 mg/L PEG, 1 g/L SPS, and 5 mg/L oligomers: (**a**) without leveler; (**b**) IPIET; (**c**) IPIEP; (**d**) IPIMP; (**e**) PIEP.

Figure 3 shows the cross-sections of the plated TH PCBs obtained from different baths. Without the leveler, the throwing power (TP) of TH is only 55.8% on average. With the 5 mg/L addition of oligomers, the TP from IPIEPT, IPIEP, IPIMP, and PIEP rises to 67.6%, 84.6%, 78.5%, and 72.1% respectively (Figure 3). According to the outcome of electroplated THs with varied oligomers, IPIEP is the preferred leveler in comparison to the other ones. The variance in the TP values demonstrates that the four oligomers, synthesized by linker groups and five/six-membered heterocycles, all possess the ability to improve the uniformity of THs plating. The copper thicknesses of the sidewall center dramatically increase (Figure 3b–e), which verifies that the four oligomers generally improve the electrical reliability of TH.

Except for PIEP, the TP values follow the order: IPIEP > IPIMP > IPIET. This sequence implies that, compared with the ethoxy and carbonyl group, the hydroxyl group is more effective in enhancing the leveling performance of the oligomers. As distinguished from the electrochemical behaviors of PIPE (Figure 1), the TP value from PIEP is higher than that from the blank. This difference possibly originates from the inhibition of the activity of accelerators and occlusion of the deposition of copper ions, which are caused by the electrostatic pairing between the cationic amine functionalities of PIEP and the negatively charged sulfonates of MPS [28].

**Figure 4:**
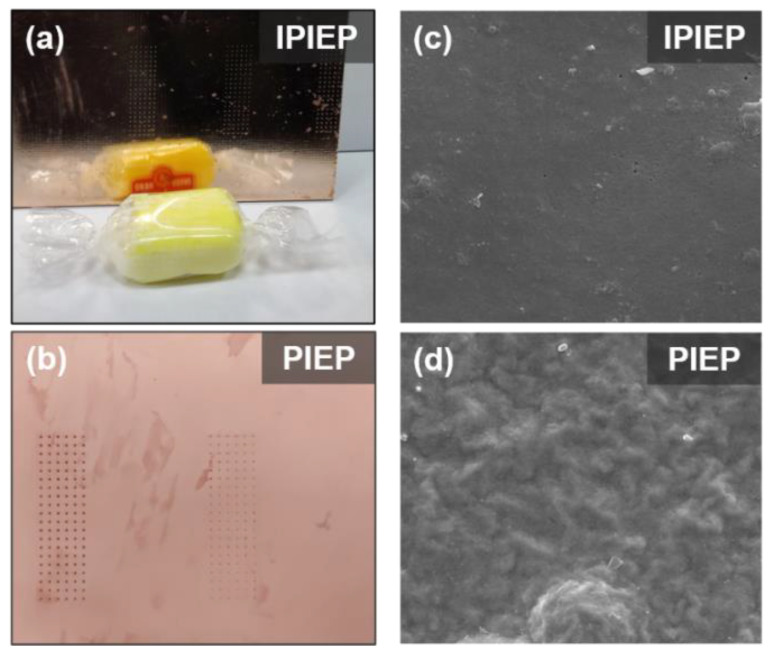
The surface appearance of the deposited copper was obtained from IPIEP (**a**) and PIEP (**b**). FE-SEM images of copper deposits obtained in the electrolyte containing: (**c**) IPIEP; (**d**) PIEP.

The appearances and SEM images of the surface copper films obtained from IPIEP and PIEP bathes are shown in Figure 4. Combing with Figure S3, IPIET, IPIEP, and IPIMP effectively smooth the roughness of the deposited layer. In a macroscopic view, the sample with the addition of IPIEP yields a mirror-like copper surface with visible reflection (Figure 4a,c). However, the injection of PIEP results in a mist appearance because its surface morphology is corrugated (Figure 4b,d), which is probably caused by the adsorption conformation (detailed discussion in Section 3.3) of PIEP.

**Figure 5:**
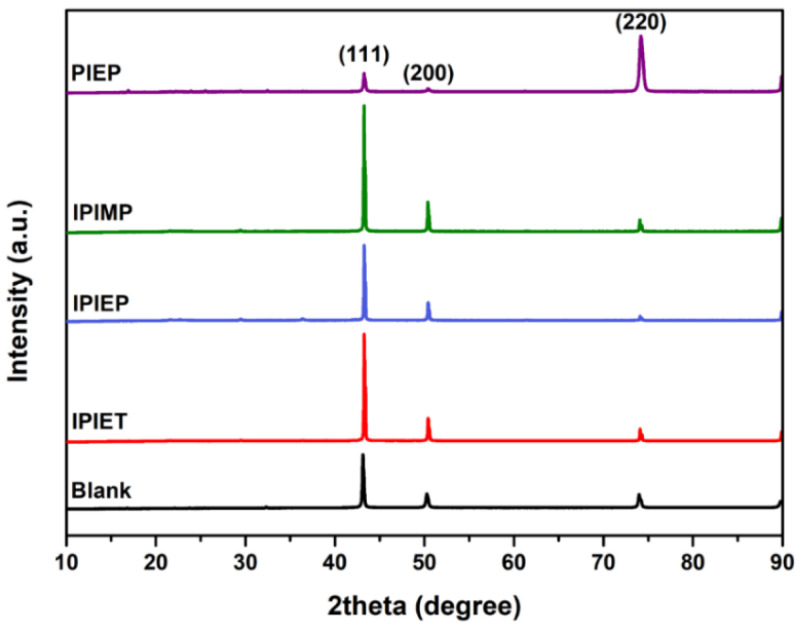
XRD patterns of the electroplated copper film obtained in the base electrolyte without or with 5 mg/L oligomers.

XRD is invited to evaluate the correlation between the branched functional groups of the levelers and the crystalline orientation of the electrodeposited copper. As shown in Figure 5, the (111) facet is still the dominant orientation of the copper films with the addition of IPIEP, IPIET, and IPIMP, which is similar to the blank sample. In contrast, after the addition of PIEP, the intensity of the diffraction peaks corresponding to the (111) and (200) planes of the copper film decreased significantly, and the (220) plane became preferred. The diverged pattern of diffraction peaks is attributed to the adsorption properties of the additive molecules at specific crystal planes [29]. The reason for the increased intensity of the (220) peak may relate to the adsorption of PIEP on the (111) and (200) faces, and effectively blocks the deposition of copper on these facets. Furthermore, the similarity in the XRD patterns that include IPIET, IPIEP, and IPIMP reveals that the branched functional group is not the decisive factor dominating the microstructure of the electrodeposited copper.

### 2.3. Quantum Chemical Calculations

**Figure 6:**
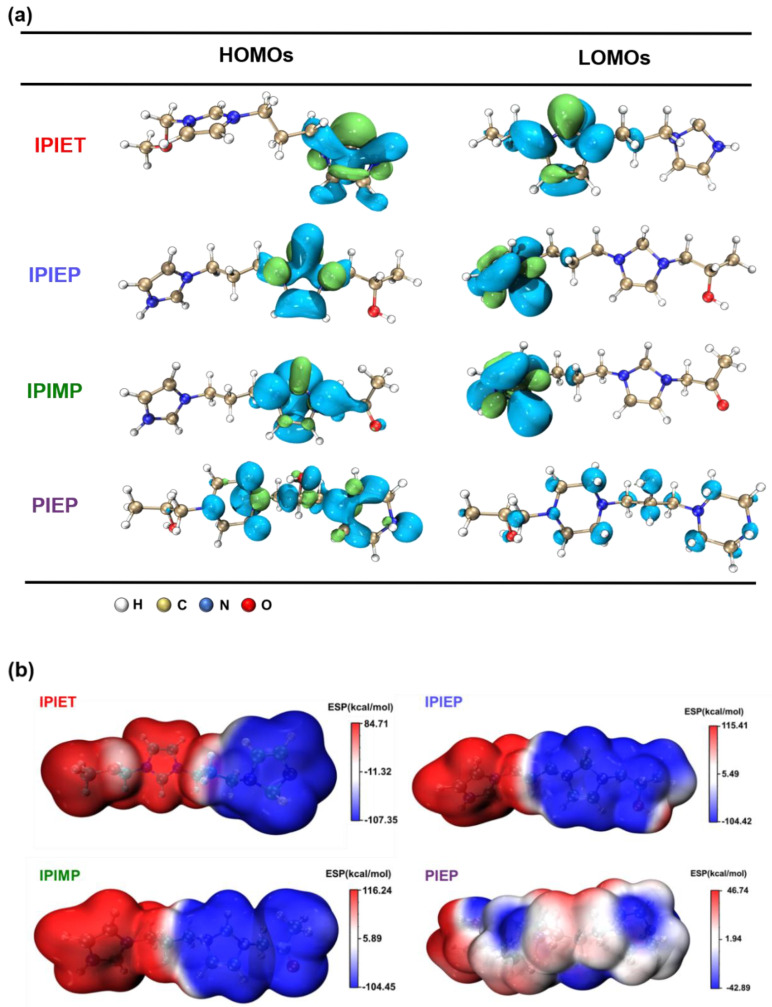
(**a**) Distributions and orbital energy values of the HOMO and LUMO for the four oligomers. (**b**) The ESP maps of the four oligomers.

Figure 6a shows the electron distributions of the HOMOs and LUMOs from molecular models of the four oligomers, with the E_HOMO_, E_LUMO_, and ΔEg (ΔEg = E_LUMO_ − E_HOMO_) given in Table 1. The results of the DFT calculations support that IPIEP is a favorable leveler th\t is beneficial from its lowest ΔEg. According to the frontier molecular orbital theory, the higher E_HOMO_ and lower E_LUMO_ represent a better electron donation and acceptance capacity, respectively. The adsorption capacity of organic additives on the high current density area of the cathode strengthens with an increasing E_HOMO_ or decreasing E_LUMO_ [30]. It is then implied that reducing ΔEg favors the adsorption capacity of levelers on the high-current-density region of the cathode. The HOMO-LUMO bandgap values of IPIET, IPIEP, and IPIMP are all below that of PIPE, following the order: ΔEg_PIEP_ > ΔEg_IPIET_ > ΔEg_IPIMP_ > ΔEg_IPIEP_. This trend leads to the deduction that the adsorption capacity of PIEP is weaker compared with the other oligomers. The order of the ΔEg values of the oligomers, except for PIEP, is consistent with their order in the cathodic steady potential (Figure 1) and TP values (Figure 3), which indicates that hydroxyl groups are the preferred branched groups for high-performance levelers.

The differences in the HOMO-LUMO bandgap, ESP value, and adsorption configuration between IPIEP and PIEP illustrate that the imidazole group enhances the performance of the leveler more effectively than piperazine. In the model, the high electron density is favorable for the occurrence of electrophilic reactions [31] and, conversely, the low electron density area is favorable for the occurrence of nucleophilic reactions [31]. Owning to the saturated heterocyclic chemical structure of piperazine, the low-electron-density regions of PIEP are distributed on the N atom of the piperazine ring, as displayed in ESP plots (Figure 6b). Due to the conjugated large π-bonds of imidazole, the low-electron-density regions of the oligomers (IPIET, IPIEP, and IPIMP) are concentrated throughout the imidazole ring. These results evidence that the electrostatic coupling of PIEP with the negatively charged sulfonate of MPS adsorbed on the copper surface is much weaker than that of the other three oligomers.

Figure S4 shows the final adsorption conformations of IPIEP and PIEP on the copper surface. Owning to the LUMO orbitals and the high-charge-density regions distributed on the imidazole ring, IPIEP holds a much higher adsorption energy (−210 kcal/mol) than PIEP (−39 kcal/mol) (Table S1). The decrement in adsorption energies implies that the ability of the cathodic polarization of IPIEP is higher than that of PIEP, which is also consistent with their electrochemical behaviors in the potentiodynamic polarization (Figure 2). Moreover, compared with the chair-like conformation of piperazine, the planar conformation of imidazole enhances the capacity of the leveler to block the deposition of copper ions more effectively, which is also reflected in the surface appearance of the copper layer (Figure 4a). All of the results of the DFT calculation and molecular dynamics (MDs) point to the conclusion that imidazole is the favorable functional group than piperazine.

### 2.4. N-Heterocyclic Oligomers on the Copper/Electrolyte Interface

**Figure 7:**
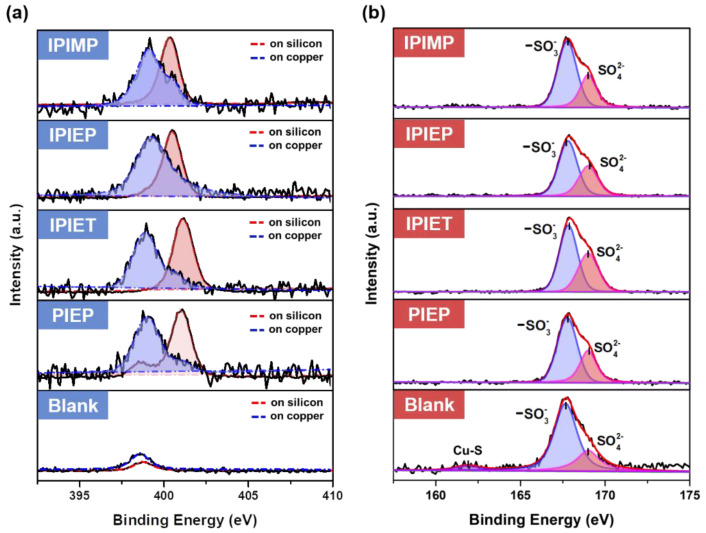
High-resolution XPS spectra of samples: (**a**) N1s peaks of oligomers on the silicon wafer and cathodic copper surface; (**b**) S2p peaks of oligomers adsorbed on the cathode.

The interaction of oligomers and the copper surface was examined by conducting surface high-resolution XPS on the cathodes to analyze the valance changes of representative elements (Figure 7a,b). The XPS of the bath solutions, which were directly dried on the silicon wafer, was used for comparison (Figure 7a). N1s spectra in Figure 7a are disintegrated into two major peaks, with the binding energy peaks at around 399 eV and 401 eV. Query from NIST’s XPS database, the N1s binding energy of quaternary ammonium salts, imidazole, and the Cu-EDTA complex is 401.6, 401.1, and 399.7 eV, which were used as the reference [32,33,34]. Figure 7a shows that these four oligomers on silicon, which present higher binding energy peaks, are probably quaternary ammonium salts due to the strong acidic chemical environment. However, their N1s peaks shift toward the lower binding energy direction of all four oligomers on copper surfaces. Two views from the former works can expend the lower N1s binding energies of oligomers on the copper substrate. The formation of the coordinate bind between N and copper, as well as the chemical adsorption of oligomers on copper surfaces, may lower the binding energy of N1s [34]. The red shift of the binding energy of the Cu-N bond in the Cu(II)-EDTA complex indicates that the coordination decreases the binding energy of the Cu−N bond [32]. On the other hand, in Schmidt’s theory, the negatively charged sulfonate of MPS pairing to the cationic N decreases the binding energy [28]. Both of the above two mechanisms explain the desorption of MPS on the copper surface and reduce the local depolarization.

An analysis of sulfur valence at the interface helps to determine the interfacial mechanism of the interaction between the leveler and accelerator. Without the leveler, a binding energy peak at 161.8 eV is spotted in the spectrum of the blank (Figure 7b). This low-valence S can be attributed to the adsorption of thiol-groups on the surface of copper [35], but this peak is invisible when the levelers are engaged. The four curves with oligomers in Figure 7b are separated into two peaks, where the peaks at 169 eV belong to sulfate on the copper surfaces [36]. According to the literature and the possible chemical components in the interface, the peak at 167.6 eV in the blank of Figure 7b belongs to S in the sulfonate of MPS [36]. When oligomers are in the interface, the binding energies of S in sulfonate shift slightly toward the higher binding energy direction in all other curves as shown in Figure 7b, indicating that the negative charge on sulfonate migrates by attraction. Therefore, the XPS analysis of S proves that, although MPS is desorbed from the copper surface after the addition of levelers, MPS remains in the interface area by pairing with positive charges from the cationic N of oligomers. This is consistent with our electrochemical experiments and Schmidt’s theory [28].

### 2.5. Mechanism

**Figure 8:**
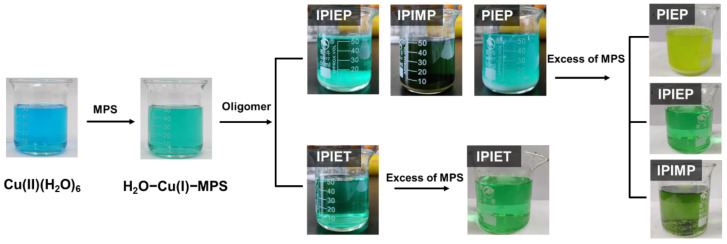
Series of photos demonstrating the coordination reactions between oligomers, Cu(I), and MPS.

In order to completely reveal the structure–performance relationship and the interfacial mechanism of the leveler, together with the interaction between the leveler and accelerator, beaker experiments were conducted. Figure 8 shows the solution reaction containing these oligomers with MPS. When oligomers with hydroxyl (IPIEP) or carbonyl (IPIMP) groups are added to the solutions, precipitations are observed, which is similar to the phenomenon described in ref. [9], which suggests that these precipitations are complexes of IPIEP−Cu(I)−MPS and IPIMP−Cu(I)−MPS. Later, these dissolve with excess MPS. In contrast, PIEP−Cu(I)−MPS remains as precipitation with excess MPS, probably due to the formation of inner salts with strong electrostatic coupling between the cationic amine of PIEP and the anionic sulfonate of MPS [9]. Moreover, the injection of IPIET into the MPS-containing solution does not lead to any sensible reaction. Combined with our electrochemical test and electroplating results, the agglomeration between H_2_O−Cu(I)−MPS and the oligomers (IPIEP, IPIMP, and PIEP) deactivate the depolarization of accelerators during the electrodeposition.

**Figure 9:**
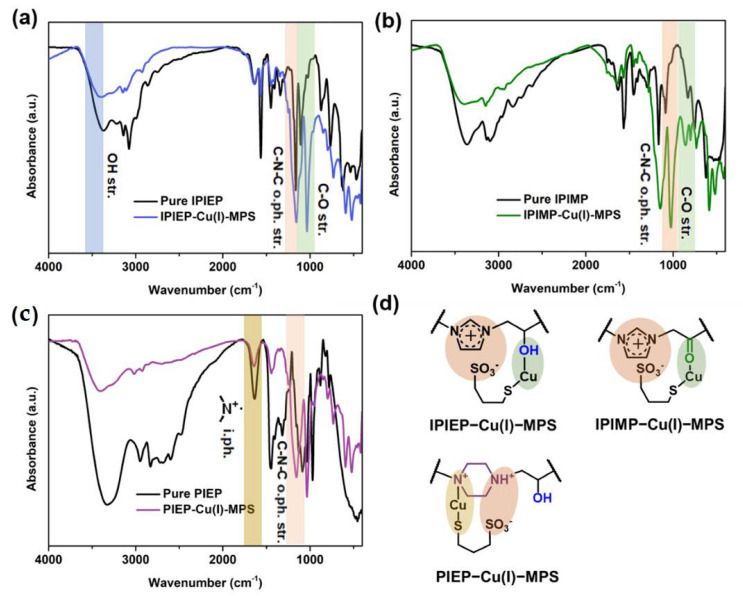
IR spectra of oligomer−Cu(I)−MPS: (**a**) PIEP, (**b**) IPIMP, and (**c**) IPIEP. (**d**) The schematic diagram of the oligomer−Cu(I)−MPS adducts.

For further investigation, the precipitations of the oligomer−Cu(I)−MPS were filtrated and characterized. The FT-IR spectrum of IPIEP, IPIMP, and PIEP and their depositions are plotted in Figure 9. In Figure 9a, the peak of C−N−C out-of-phase stretching at 1164 cm^−1^ is weakened and redshifted, indicating that the imidazole ring is pulled and stretched. The blue shift of the peaks of C−O stretching and −OH stretching increases the electron density of the oxygen, which implies the existence of oxygen coordination bonds. The chemical structure of IPIEP−Cu(I)−MPS is drawn in Figure 9d, where the charge interaction and the coordination fit the peak shift trends in the IR spectrum. The situation of IPIMP is similar in Figure 9b, and the chemical structure is also drawn in Figure 9d. However, it is observed that the peaks of C−O stretching and C−N−C out-of-phase stretching both shift to the higher wavenumber bands (Figure 9c), indicating that the interaction between PIEP and H_2_O−Cu(I)−MPS is different from IPIEP and IPIMP. Moreover, the significantly decreased intensity of the R_4_N^+^ peak at 1636 cm^−1^ indicates that the complex of IPIEP and H_2_O−Cu(I)−MPS is similar to the reported polyethyleneimine (PEI), owing to its multiple cationic amine functionalities [9].

Although the DFT calculation results reveal that the bond length of Cu−O in IPIMP−Cu(I)−MPS is shorter than that of IPIEP−Cu(I)−MPS (Figure S5), the overall energy of IPIEP−Cu(I)−MPS is lower than that of IPIMP−Cu(I)−MPS (Table S2). The lower total energy of IPIEP−Cu(I)−MPS implies that it is more stable, which means that the hydroxyl group is a favorable branched functional group for high-performance levelers. The consistency between the electrochemistry analysis (Figure 1), electroplating THs (Figure 3), and beaker experimental results demonstrate the synergistic effect of the molecular skeleton structure, positively charged functional groups, and branched groups for the leveling performance.

**Figure 10:**
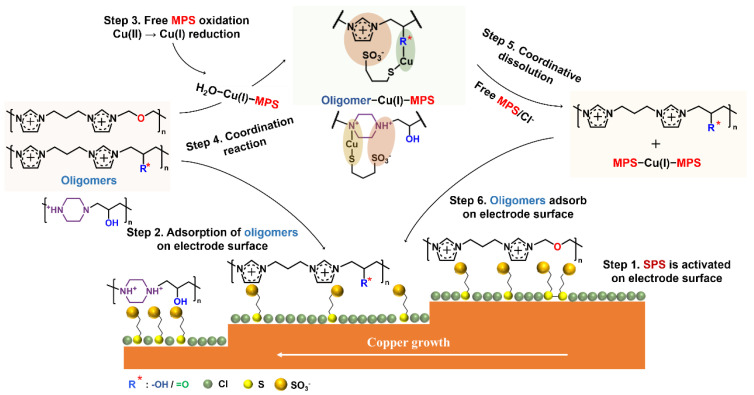
Schematic diagram of N-heterocyclic oligomers action mechanism in the bath.

The conventional mechanism of the levelers’ behavior proposes that the levelers directly adsorb on the tips or protrusions of the cathode to inhibit the deposition of metal ions [37]. Schmidt found that PEI deactivates MPS to achieve leveling via the electrostatic pairing between the cationic amine and the negatively charged sulfonate of SPS on the copper surface [28]. The beaker experiment from Broekmann advises that the hydroxyl groups of IMEP overlay a secondary suppression to MPS [27]. Combining these theoretical models and the experimental results of the four N-heterocyclic oligomers in this work, we propose the mechanism of these levelers in the plating bath (Figure 10).

The proposed model of this work assumes that SPS acts as a precursor for the monomeric MPS, which is the actual accelerator [38]. Firstly, the free SPS competes with the chloride ions to adsorb on the copper surface (step 1 in Figure 10). After replacing chloride ions, the adsorbed SPS gains electrons and decomposes into MPS. Then, the adsorption–desorption between free MPS and adsorbed MPS reaches equilibrium at the copper/electrolyte interface. The adsorbed MPS captures the dehydrated copper ions and passes them to the chloride ions to accelerate the reduction of copper [9,38]. With the addition of levelers, step 2 in Figure 10 demonstrates that the cationic amine groups are electrostatically paired at the copper/electrolyte interface with the negatively charged sulfonate ions of MPS [28], which is in agreement with the XPS spectra shown in Figure 7. Due to the electrostatic coupling between MPS and oligomers, the effect of MPS is weakened [39]. Hence, fewer copper ions are deposited on fast mass transportation regions (tips or protrusions), showing the micro-leveling phenomenon.

Step 3 in Figure 10 presents the formation of H_2_O−Cu(I)−MPS, where the free MPS capture reduced Cu(I) by coordination. According to our beaker experiment results, IPIEP, IPIMP, and PIEP coordinate with H_2_O−Cu(I)−MPS (step 4 in Figure 10). Owing to the extra negative charge from the sulfonate part, the cationic amine of IPIEP and IPIMP are electrostatically coupled with MPS to form the ‘inner salt’ in the forms of IPIEP−Cu(I)−MPS and IPIMP−Cu(I)−MPS, thus increasing the overall hydrophobicity of the ensembles [9]. The single cationic amine groups on IPIEP and IPIMP also form the oligomer−Cu(I)−MPS adducts through inter- and intra-chain electrostatic pairing. On the other hand, IPIEP−Cu(I)−MPS and IPIMP−Cu(I)−MPS change back into a solute with excess complexation ligands such as chloride or free MPS (step 5 in Figure 10). Step 6 shows the “idle” IPIEP and IPIMP absorbed on the cathode surface or coordinating with the H_2_O−Cu(I)−MPS in the electroplating solution. The reaction cycle (steps 2–6 in Figure 10) of IPIEP and IPIMP helps the plating solution achieve a higher uniformity of THs (Figure 3c,e).

The DFT results by Simona et al. [40] suggest that the coordination of N−Cu(I)−S is more stable than that of O−Cu(I)−S, which is the key to the formation of IMEP−Cu(I)−MPS. It is also reasonable to explain the electroplating behavior of PIEP based on the similar structure of multiple cationic amines of the PEI [9]. The deactivation mechanism of the PIEP−Cu(I)−MPS coordination closes a second loop in the MPS reaction cycle and expedites the consumption of MPS. Compared to the other three oligomers, IPIET does not exhibit similar plating behavior because the oxygen atom in the ethoxy group is electrostatic by the two adjacent imidazole rings, and coordination between the oxygen of ethoxy and Cu(I) is blocked.

The coordination of IPIEP−Cu(I)−MPS and IPIMP−Cu(I)−MPS presents overlay inhibition beyond the anion–cation pairing [9], which improves the uniformity of the TH plating. Nevertheless, the hydroxyl group is the preferred branched functionality for synthesizing high-performance levelers. Due to the tetrahedron configuration of sp3 hybridization and the free rotation of the C−O bond, the hydroxyl group reduces the twist of the chemical bond in the leveler−Cu(I)−MPS complex. Conversely, the rotation is more difficult for the carbonyl group in the complex because of the rigidity of the planar configuration of sp2 of C=O.

## 3. Materials and Methods

### 3.1. Synthesis of the Oligomers

**Figure 11:**
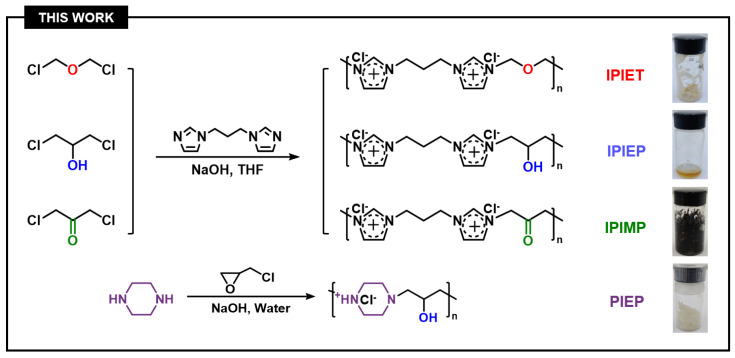
The synthesis of oligomers with varied donor units.

The four N-heterocyclic oligomers (PIEP, IPIEP, IPIET, and IPIMP) were used to investigate the structural effect of the interaction between different functional groups and other components on the performance of oligomers (Figure 11). Among these oligomers, PIEP was synthesized from piperazine and epichlorohydrin in an aqueous solution, following the same synthetic route of the IMEP [9]. The intermediate 1,3-bis(1-imidazolyl)propane was a pale yellow oily liquid, which was synthesized from imidazole and 1,3-dibromopropane in tetrahydrofuran. IPIP, IPIET, IPIEP, and IPIMP were prepared from an acetonitrile solution of 1,3-bis(1-imidazolyl)propane and their corresponding dichloromonomers. All the reactions were carried out at the reflux temperature for 8 h, then the solvents were removed and the products were dried in vacuum at 60 °C overnight. The molecular structures of PIEP, IPIET, IPIEP, and IPIMP are shown in Figure 11, and the detailed characterizations are shown in the Supporting information.

### 3.2. Characterization

The nuclear magnetic resonance spectra for ^1^H (^1^H NMR) were recorded by a Bruker Avance 400 spectrometer at a resonant frequency of 400 MHz (Bruker, Billerica, MA, USA) with deuterated chloroform (CDCl_3_) or deuterium oxide (D_2_O) as the solvent and tetramethylsilane as the references. A potentiostat (PGSTAT302N, Metrohm, Herisau, Switzerland) was used for all electrochemical measurements. Field-emission scanning electron microscopy (FE-SEM, Hitachi SU5000, Tokyo, Japan) was employed to evaluate the quality of the copper film (Section 2.2). X-ray diffraction (XRD) patterns were tested by Shimadzu XRD-7000 (Section 2.2) (Kyoto, Japan). X-ray photoelectron spectroscopy (XPS) (Section 2.4) was conducted on a Thermo Scientific^TM^ K-Alpha^TM+^ spectrometer equipped (Waltham, MA, USA) with a monochromatic Al K_α_ X-ray source (1486.6 eV) operating at 100 W.

### 3.3. Quantum Chemical Calculation

Calculations of molecular orbital information and charge distributions were performed using the Gaussian 09 programs package (Section 2.3) [20]. Geometry optimizations were conducted by density function theory (DFT) using the B3LYP theoretical method and gen basis set [41], which is 6-311G + (d, p) for all atoms [42]. Self-consistent reaction field (SCRF) theory with Tomasi’s polarized continuum model (PCM) was utilized to perform the calculations in solution [43]. After the optimization, the frontier orbitals and electrostatic potential (ESP) were examined by a visualization program to obtain the preferred reaction sites [44,45].

### 3.4. Electrochemical Experiments

All of the electrochemical measurements (Section 2.1) were operated at 25 °C by using a working electrode (a platinum rotating disk electrode with a 3 mm diameter, Pt-RDE), a counter electrode (copper rod), and a reference electrode (saturated Hg/Hg_2_SO_4_ electrode, SME), respectively [46]. The composition of the base electrolyte used for all electrochemical tests contained 100 mg/L NaCl, 75 g/L CuSO_4_·5H_2_O, and 240 g/L H_2_SO_4_. Polyethylene glycol (PEG, MW = 10,000) and SPS served as a suppressor and an accelerator, respectively. PIEP, IPIET, IPIEP, and IPIMP were employed as levelers in this work.

Galvanostatic measurements (GM) with the injection of additives were conducted with a Cu-RDE tip at different rotation speeds, and the current density was maintained at 1.5 A/dm^2^ [46]. For the potentiodynamic polarization test, the sweep ranged from −0.6 V to 0.8 V, and the rotation speed of the working electrode was fixed at 1500 rpm with a scan rate of 0.02 V/s. Before the electrochemical experiments, a Pt-RDE tip was pre-plated in the base electrolyte for 5 min to prepare the fresh and clean Cu-RDE tip.

### 3.5. Electroplating

The test samples for electroplating were 15 × 5 cm^2^ PCBs with multiple THs (Section 2.2). The diameter and depth of the THs were 0.15 mm and 1.5 mm, respectively. Two phosphorated copper plates were used as the anodes and directly placed in a Haring cell with 1500 mL electrolyte. The PCBs were electroplated at a current density of 1.5 A/dm^2^ for 70 min at 25 °C. To ensure adequate mass transportation, a continuous air bubble flow was adopted during the electroplating process. The leveling performance of the leveler was evaluated by the TP [47], whose definition and calculation are shown in supporting information (Figure S1).

### 3.6. Synthesis of Oligomer−Cu(I)−MPS Adducts

To characterize the interaction among the oligomers and other solution components (Section 2.5), the injection of Cu(I) and MPS was explored. The experiment setup referred to the beaker experiment mentioned in the reported article [9], and the adducts were characterized by Fourier transform infrared (FT-IR) spectroscopy (ThermoFisher, Waltham, MA, USA, Nicolet IS5).

## 4. Conclusions

In this work, we successfully synthesized and characterized a series of oligomers involving N-heterocyclic groups and O-functional groups via methylene links. The conformational relationship between the molecular structure design and its electroplating leveling ability was investigated. The electroplating of THs with the oligomers demonstrates (Section 2.2) that all four oligomers possess a leveling ability. The DFT calculation results suggest that the types of N-heterocyclic functionality affect the performance and electroplating behaviors (Section 2.1, Section 2.2 and Section 2.3) of the oligomers and that imidazole exhibits a better performance than piperazine for the synthesis of high-performance straight-chain levelers.

Most importantly, a chemical model (Figure 10) for the four oligomers is proposed (Section 2.4 and Section 2.5). The inhibition of MPS activity is the key factor of this model, and is caused by the electrostatic pairing between the cationic amine functionalities of oligomers and the negatively charged sulfonate of MPS. The additional inter- or intra-chain anion–cation pairing between H_2_O−Cu(I)−MPS complexes and the functional groups of oligomers also contributes to the mechanism of leveling. Among these oligomers, IPIEP is the best leveler for THs plating because the modification of the hydroxyl enhances the hydrophilicity of IPIEP and triggers a more stable coupling between IPIEP and H_2_O−Cu(I)−MPS (Section 2.5). Notably, the hydroxyl or carbonyl group is the key to initiating the coordination process between levelers and H_2_O−Cu(I)−MPS, which implies that the other O-functional groups, such as the carboxyl group and aldehyde group, will also bring the extra coupling process. Research focusing on the detailed mechanism of related additives in electroplating baths will be carried out in the follow-up work.

## Figures and Tables

**Table 1:** Energy values of molecular orbital and bandgap energy (ΔEg) of oligomers.

Oligomer	E_HOMO_ (eV)	E_LUMO_ (eV)	ΔEg (eV)
PIEP	−5.53	−0.04	5.49
IPIET	−3.73	−1.36	2.37
IPIMP	−2.94	−1.25	1.69
IPIEP	−2.83	−1.25	1.59

## Data Availability

Not applicable.

## Supplementary Materials

The following supporting information can be downloaded at: https://www.mdpi.com/article/10.3390/molecules28062783/s1, Figure S1: The schematic diagram of the through-hole; Figure S2: Cyclic voltammograms each containing 5 mg/L of oligomers in the 5% H_2_SO_4_ electrolyte: (a) PIPE; (b) IPIET; (c) IPIEP; and (d) IPIMP; Figure S3: FE-SEM photos of copper films obtained from the electrolytes containing: (a) base electrolyte; (b) IPIET; (c) IPIMP; Figure S4: (a) Molecular structure of imidazole and piperazine molecules; The final adsorption conformation of IPIEP (b) and PIEP (c) on the copper surface; Figure S5: The Cu-O bond length calculation results of different oligomer−Cu(I)−MPS: (a) IPIEP and (b) PIEP; Table S1: The adsorption energy of adsorbate IPIEP and PIEP; Table S2: The overall energy of IPIEP-Cu(I)-MPS and IPIMP-Cu(I)-MPS.

## Sample Availability

Samples of the compounds are available from the authors.
