# Supplementary material for: Structure Performance Correlation of N-Heterocyclic Oligomer Leveler for Acid Copper Plating of Advanced Interconnects
Source: Molecules. 2023 Mar 20;28(6):2783. doi: 10.3390/molecules28062783 (PMC10051102; doi:10.3390/molecules28062783)
Supplement: Supplementary file 1 [file molecules-28-02783-s001.zip › molecules-2272874-supplementary.pdf]

Supporting Information for *“Structure Performance Correlation of N-heterocyclic Oligomer Leveler for Acid Copper Plating of Advanced Interconnects”*

Synthesis of Levelers:

General Procedure for the Synthesis of Compound PIEP

Piperazine (0.8614 g, 0.01 mol) was dissolved in 5 ml of water and stirred at room temperature for 1 h, and then epichlorohydrin (0.9252 g, 0.01 mol) was slowly injected dropwise into the reaction device. After one hour, the reaction temperature was raised to 85°C and stirred at this temperature overnight. After the reaction was stopped, the reaction solution was cooled to room temperature and dried in a vacuum to remove the solvent. The remaining solid was washed with ethanol three times and dried in vacuo at 60° C overnight to obtain 1.1613 g of a faint yellow solid (yield: 65%).

General Procedure for the Synthesis of Compound IMEP

Imidazole (13.62 g, 0.2 mol) was dissolved in 100 ml of water and stirred at room temperature for 1 h, and epichlorohydrin (18.50 g, 0.2 mol) was slowly added dropwise into the reaction device to continue stirring. Then, the reaction temperature was raised to 85°C and stirred at this temperature overnight. After the reaction was stopped, the reaction solution was cooled to room temperature and dried in vacuo to remove the solvent. The remaining solid was washed with ethanol three times and dried in vacuo at 60°C overnight to obtain 30.51 g of a faint yellow solid (yield: 95%).

Procedure for the Synthesis of Compound 1,3-Bis(1-imidazolyl)propane

After imidazole (13.62 g, 0.2 mol) was dissolved in 25 ml of tetrahydrofuran at room temperature with stirring, sodium hydride (0.22 mol)

was added to the solution in batches, and the solution was stirred until no bubbles appeared. Then, 1,3-dibromopropane (18.17 g, 0.09 mol) was slowly added dropwise to the reaction device, and the reaction was stopped after stirring at room temperature for 48 hours. The mixed solution was suction filtered, and the filtrate was collected and dried under a vacuum to finally obtain 15 g of a faint yellow oily liquid (yield: 42.62%).

General Procedure for the Synthesis of Compound IPIP, IPIET, IPIEP, IPIMP.

Polymers IPIP, IPIET, IPIEP, and IPIMP were prepared in the same synthetic routes, and only the preparation process of IPIP is described in detail here. 1,3-bis(1-imidazolyl)propane (0.8811 g, 0.005 mol) was dissolved in 5 ml of acetonitrile and stirred at room temperature for 1 hour, and then 1,3-dichloropropane (0.5650 g, 0.005 mol) was slowly added dropwise to the reaction. Stirring was continued in the apparatus and, after 1 h, the reaction was warmed to reflux and stirred at this temperature overnight. After the reaction was stopped, the reaction solution was cooled to room temperature and dried in a vacuum to remove the solvent. The remaining solid was dissolved in water and washed three times with ethyl acetate. The aqueous phase was collected and dried in vacuo at 60°C overnight to obtain 1.0122 g of a faint yellow solid. (Yield: 70%)

1,3-Bis(1-imidazolyl)propane: 1H NMR (CDCl3, 25 ℃, 300 MHz): δ 1.87-1.91 (t, -CH-, 1H), 3.36-3.51 (d, imidazole $-CH_2-$, 4H), 6.53-7.05 (s, imidazole-H, 3H). 13C NMR (CDCl3, 25 ℃, 300 MHz): δ 31.68, 43.32, 118.63, 129.51, 136.89.

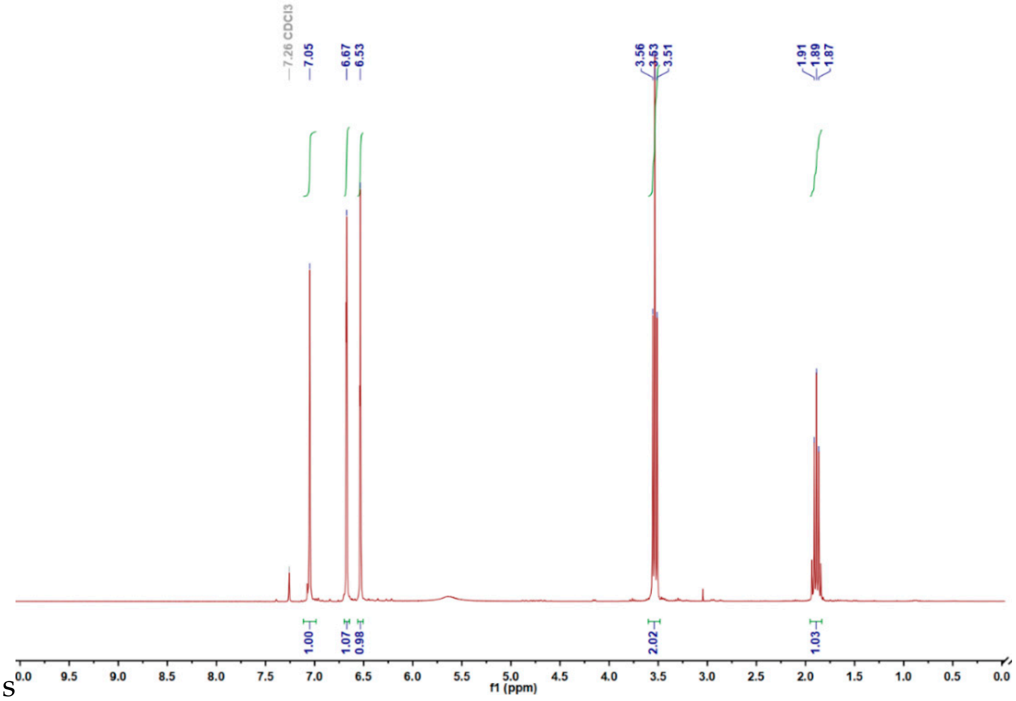

s

PIEP: ^{1}H NMR (D_2O, 25 ℃, 300 MHz): δ 2.59-3.31 (piperazine-H, $-CH_2-$), 4.31 (-CH-).

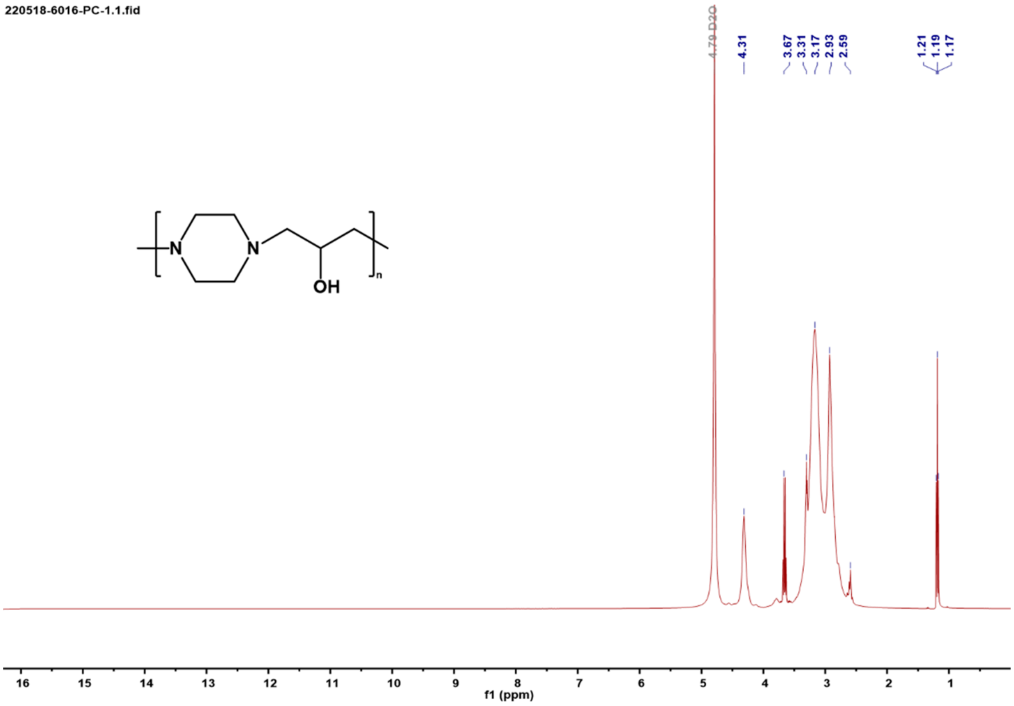

IPIET: ^{1}H NMR (D_2O, 25 ℃, 300 MHz): δ 2.32-2.57 (-CH_2-), 3.92-4.47 (imidazole-CH_2-, -CH_2-O-CH_2-), 6.99-7.60 (imidazole-H).

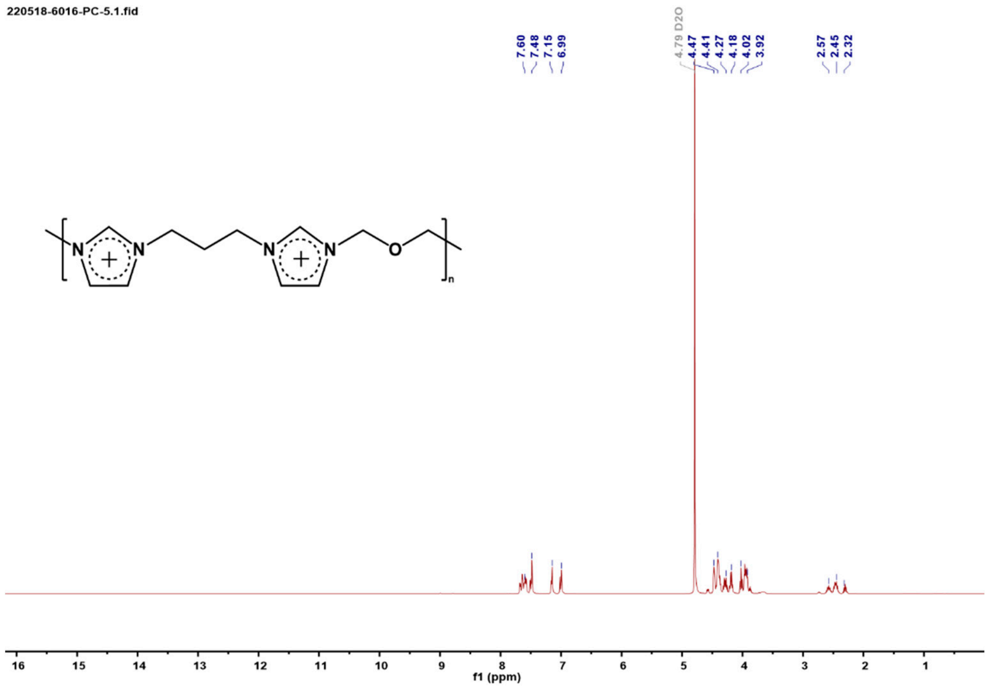

IPIEP: ^{1}H NMR (D_2O, 25 ℃, 300 MHz): δ 2.62 (-CH_2-), 4.32-4.65 (imidazole-CH_2-, -CH-), 7.34-9.04 (imidazole-H).

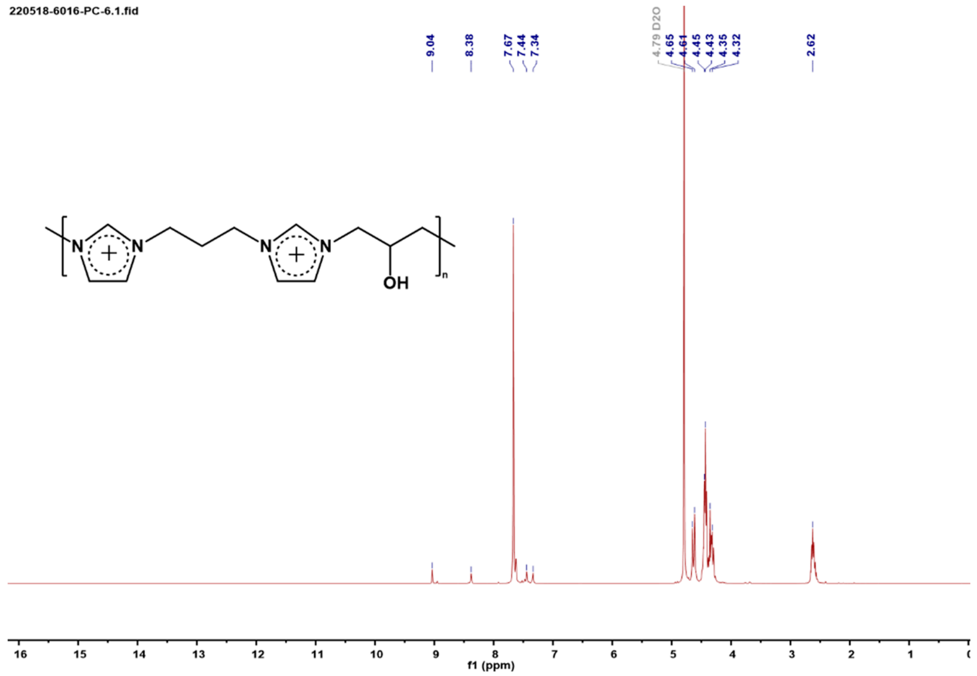

IPIMP: ^{1}H NMR (D_2O, 25 ℃, 300 MHz): δ 2.63 (-CH_2-), 4.40-4.50 (imidazole-

CH2-, -CH-), 7.53-9.02 (imidazole-H).

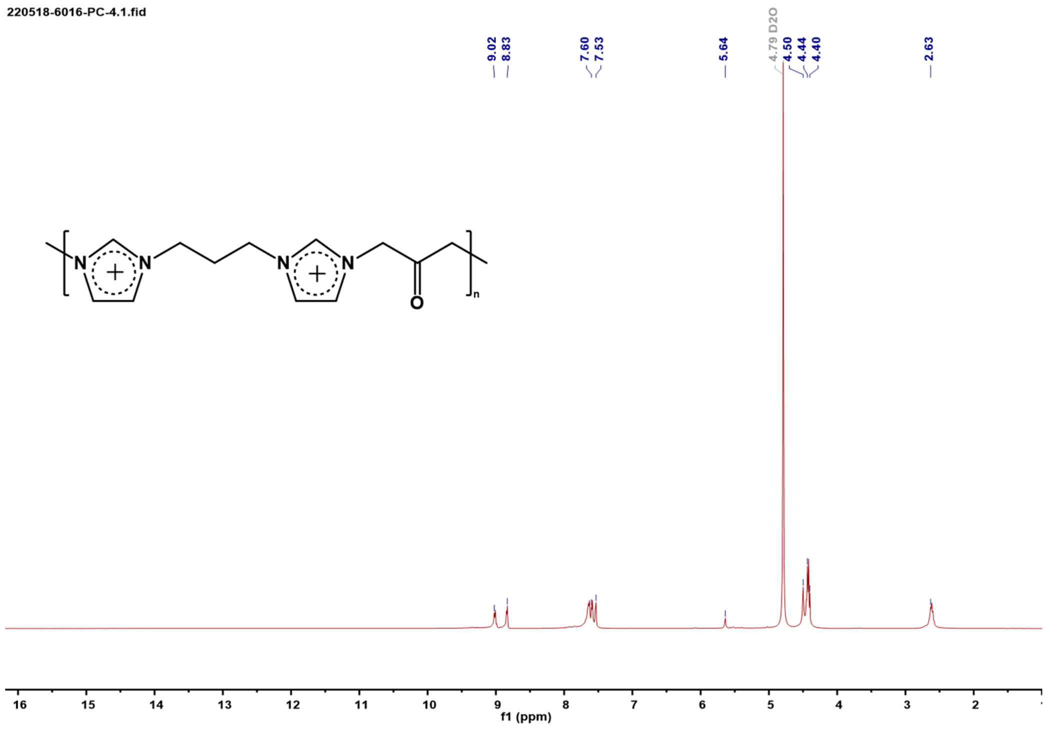

Throwing power (TP) is the most important indicator for the leveling performance of the leveler, and is calculated by Equation (1). H_a, H_c, H_d, and H_f present the copper thickness on the surface, whereas H_b and H_e mean the copper thickness in the center as shown in Figure S1.

$$\mathbf{TP} = \frac{2(H_b + H_e)}{H_a + H_c + H_d + H_f} \times 100\% \qquad (1)$$

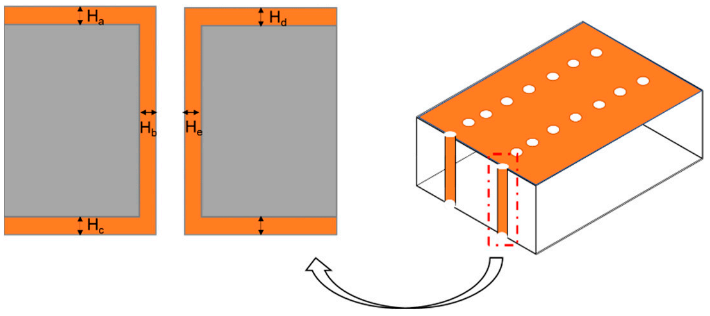

Figure S1. The schematic diagram of the through-hole.

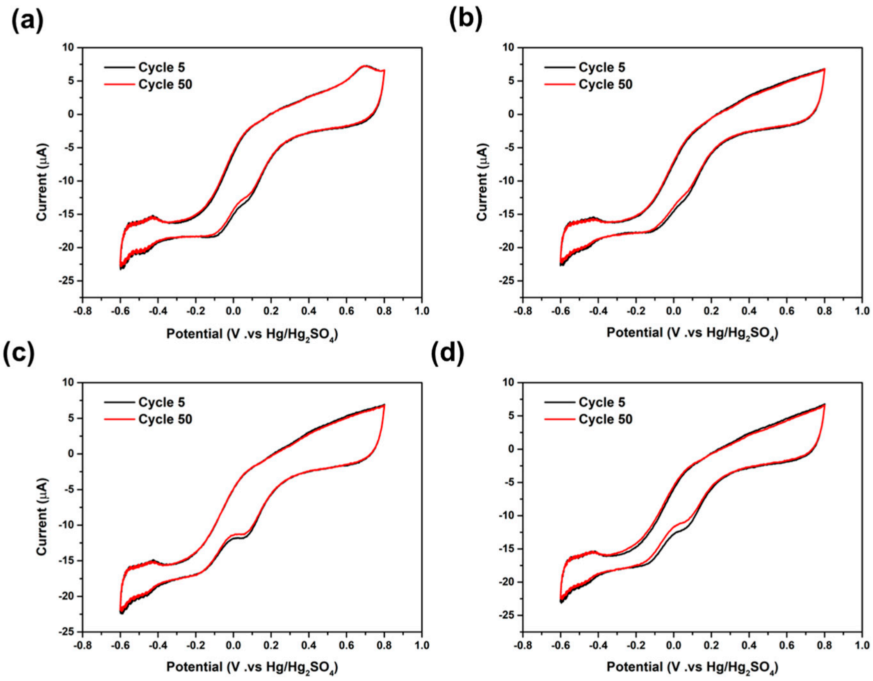

Figure S2. Cyclic voltammograms each containing 5mg/L of oligomers in the 5%H_2SO_4 electrolyte: (a) PIPE; (b) IPIET; (c) IPIEP; and (d) IPIMP.

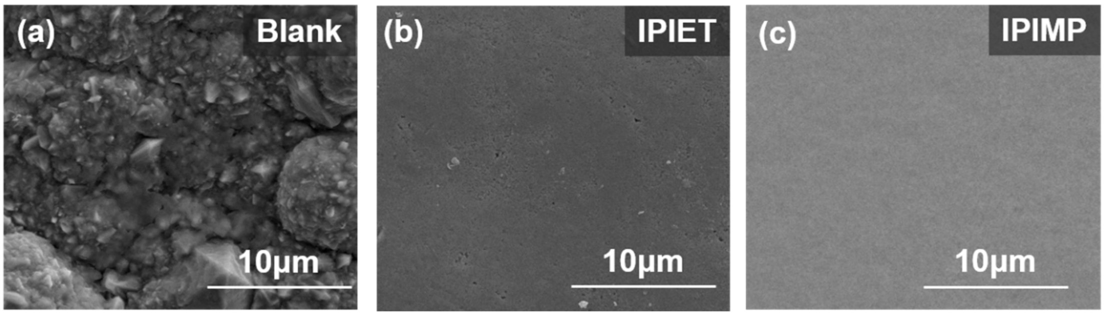

Figure S3. FE-SEM photos of copper films obtained from the electrolytes containing: (a) base electrolyte; (b) IPIET; (c) IPIMP.

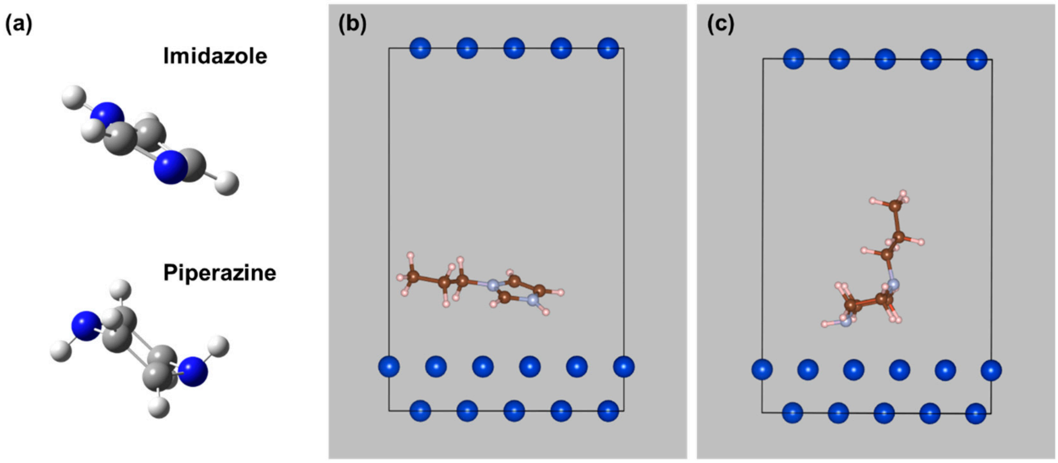

Figure S4. (a) Molecular structure of imidazole and piperazine molecules. The final adsorption conformation of IPIEP (b) and PIEP (c) on the copper surface.

Table S1. The adsorption energy of adsorbate IPIEP and PIEP.

Oligomer	$E_{additive+Cu}$ (eV)	$E_{additive}$ (eV)	E_{Cu} (eV)	E_{ads} (eV)	E_{ads} (kcal/mol)
IPIEP	-274.54	-108.87	-163.49	-2.18	-210
PIEP	-302.18	-138.28	-163.49	-0.41	-39

The adsorption energy of adsorbate oligomers is defined as:

$$E_{ads} = E_{additve+Cu} - (E_{additive} + E_{Cu})$$

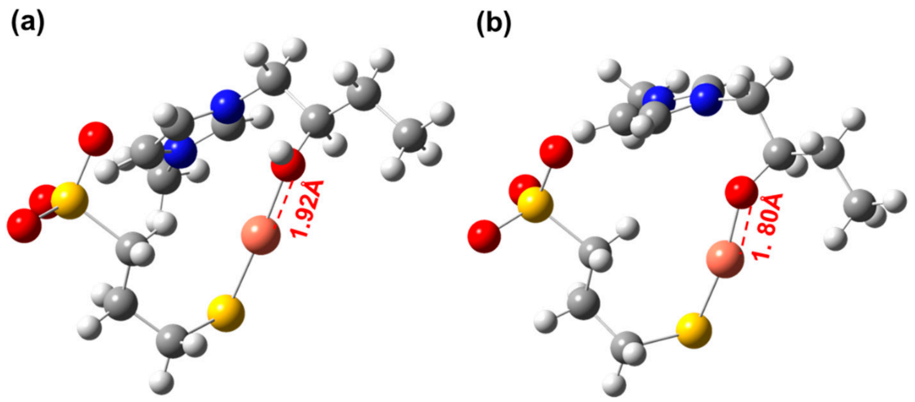

Figure S5. The Cu-O bond length calculation results of different oligomer–Cu(I)–MPS: (a) IPIEP and (b) PIEP.

Table S2. The overall energy of IPIEP-Cu(I)-MPS and IPIMP-Cu(I)-MPS.

Oligomer	$E_{overall}$ (Hartree)
IPIEP	-1058.26
IPIMP	-1057.56
